# Endoscopic Thyroidectomy for Differentiated Thyroid Cancer

**DOI:** 10.1100/2012/456807

**Published:** 2012-12-11

**Authors:** Yi Yang, Xiaodong Gu, Xiaoxiao Wang, Jianbin Xiang, Zongyou Chen

**Affiliations:** Department of General surgery, Huashan Hospital, Fudan University, Shanghai 200040, China

## Abstract

Endoscopic thyroidectomy is a relatively new approach in treating differentiated thyroid cancer. Since last decades, more and more endoscopic thyroidectomies have been performed. We review the indications and contraindications, methods, and postoperational complications of this surgical procedure. Lots of surgical approaches have been developed in endoscopic thyroidectomy for differentiated thyroid cancer. Compared to conventional thyroidectomy, scarless endoscopic thyroidectomy has a superior cosmetic result. And it also reduces the incidence of hypesthesia, paresthesia, or feelings of self-consciousness. However, the outcome depends, to a large extent, on the skill of the operator and the learning curve being relatively long. With the development of surgical equipments and skills, operation time and complications will be reduced. Indications of endoscopic thyroidectomy will be widened and it will be more and more performed.

## 1. Introduction

Differentiated thyroid cancer is the most common malignancy of the endocrine system, and the treatment develops rapidly in these decades. Conventional thyroidectomy has been a mature, safe, and efficacious treatment for differentiated thyroid cancer for the past few decades, but one incisional scar left in the anterior lower neck cannot be avoided. The scar results in a permanent cosmetic defect, which affects the aesthetic outcome [1]. And also, such a scar can lead to cervical hypesthesias, paresthesias, and feelings of self-consciousness, as the shortcoming of conventional thyroidectomy [2].

The evolutionary development of laparoscopic surgery satisfies the esthetic demand, recovery, and limited trauma in nearly all fields of surgical disciplines, including the treatment of differentiated thyroid cancer. Gagner [3] reported the first endoscopic neck surgery in 1996. And video-assisted thyroid lobectomy by Hüscher et al. [4] was operated in 1997. Soon, endoscopic thyroidectomy (ET) was developed into scarless operation by Ikeda et al. [5] and Ohgami et al. [6], by using alternative techniques. Miccoli et al. [7] did invasive video-assisted thyroidectomy for papillary carcinoma successfully in 2001. Various methods of scarless endoscopic thyroidectomy (SET) procedure were subsequently introduced in the following decade. The applicability of ET for thyroid cancer was investigated in several studies [8]. This kind of operation has attracted worldwide attention and interest on account of the high cosmetic satisfaction and less shortcomings.

## 2. Advantages and Disadvantages

### 2.1. Advantages

Compared to conventional thyroidectomy, SET has a superior cosmetic result. Jiang et al. [9] confirmed the conclusion that SET is cosmetically advantageous over open thyroidectomy. ET also provides alternative benefits of significantly reducing the incidence of hypesthesia or paresthesia and discomfort while swallowing. Moreover, the postoperative pain of SET is comparatively less severe. According to Jiang's research, the severity of postoperative pain on the first day in the conventional group was significantly higher than that in endoscopic group, but the difference was not distinguished after 48 hours.

### 2.2. Disadvantages

SET is a technically challenging procedure and the learning curve is very long. Del Rio et al. [10] reported their learning curve for minimally invasive video-assisted thyroidectomy. The mean operating time for the first 50 cases (100 cases in total) was significantly higher than the mean operation time for the remaining 50 cases. Tan et al. [1] summarized the recent articles and come to a conclusion that SET (through axilla or breast) is not a surgery of minimally injury, but only scarless in the neck. It has a longer operation time, more postoperational pain, and more severe injury than conventional procedure. There is still a risk of conversion to open surgery in certain patients who undergo ET. Arterial bleeding and large tumor size are both difficult to deal with under endoscopic procedures in comparison to open surgery. And Geng-Zhen et al. [11] reported that the conversion rate was 2.21% in the cervical group, higher than that in extracervical group (0.42%). On the other hand, there is still limited evidence provided by multicentral, big sample, prospective studies that SET can provide a satisfied recurrence rate.

## 3. Indications and Contraindications

### 3.1. Indications

The advantages of surgery performed by using minimally invasive techniques have been documented. As well, this kind of surgery has been introduced into the field of the differentiated thyroid cancer treatment for certain features, such as improved visualization and excellent cosmesis. In the early age, ET was only used for benign tumor of thyroid gland and malignant tumor was considered as one of the contraindications [12]. With the development of operation instruments and personal skills, the indications for ET are being extended and consummated continually. Different reports have shown variable indications of ET for thyroid cancer these years. 

#### 3.1.1. Classification for the Types of Operation

According to the types of operation, ET can be divided into video-assisted thyroidectomy (VAT) and total endoscopic thyroidectomy (TET). VAT works with an incision length of 1.5–2.0 cm in the anterior lower neck and gasless lifting system, while TET, except of transsupraclavicular approach (tiny scar in the neck available), is also called SET [13]. VAT has been widely accepted as a minimally invasive procedure, whereas SET has the best cosmetic results [14].

**VAT**

Miccoli et al. [15–17] firstly used VAT for papillary thyroid carcinoma (PTC) treatment. They set the indications as follows: (1) the largest diameter of the tumor is less than 3.5 cm; (2) the volume of thyroid gland is less than 30 mL; (3) no evidence of local or remote metastasis; (4) younger than 45 years old; (5) without autoimmune thyroiditis and large thyroid nodules; (6) no clinical history of radiotherapy or surgery on the neck; (7) no hyperplastic scar on the neck; (8) no blood coagulation dysfunction, respiratory dysfunction, or heart dysfunction. Lombardi et al. [18] reported that 359 patients of PTC treated with VAT (including 285 of pT1, 26 of pT2, 48 of pT3, 126 of them took central lymph node dissection, 27 of which detected lymph node metastasis by pathology) had the same survival rate with those who took traditional operation after 10-year followup. It showed that intermediate-risk PTC can also take the treatment of VAT for chances.

**TET**

There are still not many cases of thyroid cancer operation with TET available. Kitano et al. [19] had the access of TET to those who are younger than 45 years old, with the mass less than 2 cm in diameter and no evidence of lymph node metastasis or local infiltration. Chung et al. [20] and Kang et al. [21] reported that low-risk papillary thyroid cancer can also be treated with TET via the axilla-breast approach with low recurring rate. Now the indications of TET can be summarized as (1) younger than 45 years old; (2) PTC less than 3 cm in diameter; (3) no local invasion; (4) no diffuse enlargement of lymph node, no adhesion or fixation of enlarged lymph nodes; (5) no enlarged lymph node on the other side or in superior mediastinum; (6) Patients who strongly request minimally invasive operations.

#### 3.1.2. Classification for the Types of Pathology

According to the classification for the types of pathology, thyroid cancer can be classified as PTC, follicular thyroid carcinoma (these two are identified as differentiated thyroid cancer), medullary thyroid carcinoma, and undifferentiated thyroid carcinoma. PTCs are commonly treated by ET on the condition of no lymph node metastasis or microcarcinoma [22]. It is recommended that patients with follicular thyroid carcinoma less than 5 cm in diameter should take ET operation [23].

### 3.2. Contraindications

Miccoli et al. [15–17] have listed the contraindications of ET procedure in their reports as well. The relative contraindications are considered to be: (1) cancer with thyroiditis; (2) having clinical history of radiotherapy or surgery on the neck. And the absolute contraindications are reported as (1) the largest diameter of the tumor is more than 3.5 cm; (2) the volume of thyroid gland is more than 30 mL; (3) evidence available of local or remote metastasis; (4) elder than 45 years old. And it is not suitable for ET if the thyroid cancer has multiple focuses, infiltrates to thyroid membrane or anterior jugular muscles, or lymph node metastasis.

## 4. Surgical Approaches

Since the first report of endoscopic neck surgery, an increasing number of ET techniques have been created. These techniques can be divided into two approaches depending on where the incisions are made: being relative to the neck (direct) or not (indirect) [24]. The cosmetic results of TET are superior to those of VET because the incisions over the anterior chest are small and can be modified by patient. But VAT surgery is reported less invasive than TET surgery during the procedure because VAT can give the direct path to the target mass; the approach of VAT is more familiar to surgeons, and the incisions are made to allow ordinary instruments to reach it [25]. And according to the insertion site of the surgical instruments, ET surgery can be divided into a cervical, anterior chest wall, breast, axilla, axilla-breast approach, and so on, each of which has its own advantages. The alternation of surgical approaches depends on the size of tumor, histology, the condition of lymph node metastasis, and the situation of local infiltration [26].

### 4.1. Cervical Approach

A cervical approach offers numerous advantages over the SET approaches [27]. Although the SET approaches result in no scar on the neck, they require extensive dissection to reach the thyroid basin and lead to a cervical hematoma. But one main disadvantage of cervical approach is the long duration of surgery for making working space. The main procedure can be listed as follows [28]. A 15–30 mm skin incision is performed in the middle line, about 2 cm above the sternal notch. The cervical linea alba is opened as much as possible for better vision. Two small retractors are used to retract and lift the operated lobe of thyroid gland and to laterally retract the muscles to maintain the working space. A 30-degree 5 mm endoscope is inserted through the skin incision. 

### 4.2. Anterior Chest Wall Approach

Gasless ET can be provided for the treatment of differentiated thyroid cancer. This approach using a modified flap-lifting device was first introduced by Kim et al. [29] and subclavicular approach shows less trauma than other SET approaches. The main procedure can be listed as follows [30]. A 3 cm oblique skin incision is made in a midclavicular line on the anterior chest wall. The working space is dissected underneath the platysma muscle, advancing from the incision to the thyroid area and across the medial border of the sternocleidomastoid muscle. A 5 mm trocar is inserted lateral to the skin incision. The margins of the incised opening are covered with a silastic material. A 30-degree 5 mm endoscope is inserted through the 5 mm port. 

TET can also be used for radical differentiated thyroid cancer. The procedure begins with a 1 cm long incision one-fourth the distance from the xiphoid to the sterna notch. A separating stick is used to perform a dissection of the subcutis and then make the insertion of a 10 mm trocar. 6–8 mmHg gas pressure CO_2_ insufflation is used to maintain working space. A 10 mm 30-degree endoscope is then inserted. After bilateral transversal incisions have been created one-third the distance from the nipple to the sternoclavicular joint, 5 mm and 10 mm trocars are inserted [31].

### 4.3. Breast Approach

The first case of this procedure is famous in China [32]. The main target is to avoid the scar in the neck. The brief procedure is described as follows [33]. The camera port over 1.5–2.0 cm is placed over the right parasternal region. A subcutaneous tunnel is created by using blunt dissection. Insufflation of CO_2_ is used to create working space. Two additional skin incisions are made at the upper margin of mammary areolae, followed by the insertion of one 5 mm trocar and another 10 mm trocar.

### 4.4. Axilla Approach

It is also an aim to achieve an optimal cosmetic result for axilla approach for thyroidectomy. The operative technique is performed as follows [34]. Raise the lesion-side arm to expose the axillary fossa. Make a 5-6 cm skin incision in the axillary fossa and elevated the skin flap under direct vision in the plane of the subplatysmal layer over the pectoralis major muscle from the axilla to the anterior central neck area. Dissect through the space between the sternal and clavicular head of the sternocleidomastoid muscle. Then dissect underneath the sternothyroid muscle to expose the thyroid gland. An external retractor is used to maintain working space.

### 4.5. Axilla-Breast Approach

Axilla-breast approach was first reported in 2007 [35]. The patient is placed in the supine position and the neck was extended slightly and the lesion-side arm is raised to fully expose the axilla. A 4.5-5.5 cm skin incision is made in the axillary fossa. The skin is elevated above the pectoralis major muscle under direct vision, until the anterior border of the sternocleidomastoid muscle is exposed. The working space is created through the skin incision in the axilla. The other 0.8–1.0 cm skin incision is made along the upper margin of the mammary areola on the tumor side for inserting a 10–12 mm trocar directed to the midline of the sternal notch [36]. Nowadays axillobilateral breast approach (ABBA) has been practiced in many cases. And it is reported that endoscopic thyroidectomy via BABA can provide a good symmetrical view of both thyroid lobes, a similar operative approach to conventional thyroidectomy, and a good cosmetic outcome [37].

### 4.6. Other Approaches

There are still some new approaches developed in these years. The most world-famous one should be natural orifice surgery (NOS). In 2008, Witzel et al. [38] presented the first experimental NOS approach for thyroidectomy, by using single-port access via an axilloscope. Karakas et al. [39] made another attempt by placing the scope in the lateral sublingual floor of the mouth. Wilhelm and Metzeg [40] developed a total endoscopic transoral approach for thyroidectomy, by using a three-point access sublingually and bilaterally in the vestibule of the mouth, and they made the first successful application in humans followed in 2009. The method has proved its possible superiority to alternative SET procedure for differentiated thyroid cancer.

## 5. Lymph Node Dissection

There are still arguments about the extension of lymph node dissection for differentiated thyroid cancer treatment. Although it is reported that 12–80% of differentiated thyroid cancer patients have lymph node metastasis when are diagnosed with such disease, it is not necessary to take preventive neck lymph node dissection [41]. Recently, total lobectomy of minimal radical operation extension for differentiated thyroid cancer and level VI dissection for thyroid cancer diagnosed as PTC are generally accepted. If there is evidence for clinical lymph node metastasis (cN_1_), functional lymph node dissection is the first choice. Caron et al. [42] recommended selective neck dissection, levels II and V dissection should not be routine. NCCN guideline suggests that levels II and V dissection should be operated when there is invasive cancer, clinical or evidence of levels II and V lymph node metastasis, or level III large mass metastasis available.

Lymph node dissection of ET has been reported in many cases these years. The extension of lymph node dissection of ET is similar to that of conventional thyroidectomy. Bellantone et al. [43] first reported successful central neck lymph node dissection in 2002. And then Bae et al. [44] reported sentinel lymph node biopsy and central neck lymph node dissection via an anterior chest wall approach. Kang et al. [21] reported 581 cases (410 cases of which are malignant tumor) of ET via an axilla approach (including prevented central neck lymph node dissection on the lesion side) with good operation outcome in 2009. And Lombardi et al. [45] reported 126 cases of central neck lymph node dissection (6.0 ± 4.1 lymph nodes on average). There was no recurrence from followup to 2010. There are few TET with neck dissection for thyroid cancer up to now. Hong et al. [46] operated on 57 cases of papillary thyroid microcarcinoma by TET with prevented central neck lymph node dissection, and the followup showed the equal effect and postoperative complications of conventional thyroidectomy.

## 6. Postoperative Complications

### 6.1. Complications Peculiar in ET Complications Caused by CO_2_

CO_2_ has a strong ability of diffusion. Body may obtain more CO_2_ during SET than in the abdominal surgery because the area lacking in serous membrane. With a pressure more than 6 mmHg, patients may have hypercapnia, pneumohypoderma, and mediastinal emphysema. In addition, the separation of subcutaneous tissue may cause fat liquefaction, incision dehiscence, wound infection, and ecchymosis.

### 6.2. Complications Similar to Conventional Surgery

The major complications of SET include injury to the recurrent laryngeal nerve (RLN) and parathyroid gland. The incidence of transient or permanent RLN injury differs greatly, varying from 0 to 10%. Because of the similarity between the surgical equipments used by VAT and conventional operation, they have the similar complications. The study which has a comparatively big population is reported by Raffaelli et al. [18]. Among 359 cases of VAT, 11 patients had transient paralysis of the RLN (3.1%), and 90 (25.0%) appeared transient hypocalcemia while 4 (1.1%) became permanent. No statistical significance between VAT and conventional operation. Chung et al. [20] observed 103 patients who had TET 25.2% had transient hypocalcemia while 1.0% turned permanent. Incident of transient paralysis of the RLN is 25.2%, higher than the conventional operation, but none of them became permanent. So SET is a safe alternative to open thyroidectomy.

## 7. Foreground

Since last decade, more and more experiences of performing ET have been accumulated. The cosmetic benefit is certain and it can also reduce the chance of hypesthesia and paresthesia. According to the reports, ET is safe and effective if cases are chosen properly. Many new techniques have been developed, such as robotic thyroidectomy for prefect cosmetic appearance and best effect [47]. Some medical centers confirmed the safety of robotic total and partial thyroidectomy. The advantages provided by the robotic system made minimally invasive thyroidectomy more feasible [48, 49]. Endoscopic sentinel lymph node biopsy of differentiated thyroid cancer is another area where this potential is being explored. However, there is still limited evidence provided by multicentral, big sample, and prospective studies. The concern of cancer recurrence still exists. With the development of surgical equipments and skills, operation time and complications will be reduced. Indications of ET will be widened and it will be more and more performed.
